# LCPBert: ProtBERT-based early-stage lung cancer prediction from T cell receptor beta sequences

**DOI:** 10.1016/j.isci.2026.115581

**Published:** 2026-04-01

**Authors:** Xin Yang, Yuwei Zhou, Zixuan Zhang, Huaichao Luo, Jian Huang

**Affiliations:** 1 Department of Clinical Laboratory, Sichuan Clinical Research Center for Cancer, Sichuan Cancer Hospital and School of Life Science and Technology, University of Electronic Science and Technology of China, Chengdu, China; 2 Department of Clinical Laboratory, Sichuan Clinical Research Center for Cancer, Sichuan Cancer Hospital & Institute, Sichuan Cancer Center, Affiliated Cancer Hospital of University of Electronic Science and Technology of China, Chengdu, China

**Keywords:** biological sciences, immunology, immunological methods, cancer systems biology

## Abstract

Early detection of lung cancer remains challenging due to limitations of current methods. We developed LCPBert, a deep learning framework leveraging peripheral blood T cell receptor beta (TCRβ) repertoires for early detection of lung cancer. LCPBert accurately discriminated lung cancer-associated TCRs (test AUC = 0.82). Based on LCRI (lung cancer risk index), LCPBert robustly stratified lung cancer risk in the external validation cohort: healthy donors (0.111 ± 0.058), benign pulmonary nodules (0.184 ± 0.113), lung cancer (0.296 ± 0.166; *p* < 0.001) and showed a spatial gradient from peripheral blood (0.296) to tumor tissue (0.384, *p* < 0.084). At an LCRI cutoff of 0.1465, LCPBert achieved 75% sensitivity and 70% specificity in discriminating lung cancer patients from healthy individuals. In a longitudinal cohort, LCRI elevation (Δ > 0.15) exceeding 0.30 after SBRT predicted distant metastasis (DM) in 75% of patients who developed DM. In addition, LCRI predicted lung cancer independently of age, sex, and TCR diversity (D50). LCPBert provides an accurate and non-invasive approach for early-stage lung cancer detection.

## Highlights

• Developed LCPBert leveraging TCRβ repertoire for early lung cancer detection
• LCPBert achieves strong healthy, benign nodules and lung cancer discrimination (AUC>0.8)
• Post-SBRT LCRI elevation (Δ > 0.15) above 0.30 predicts distant metastasis
• LCRI predicted lung cancer independently of age, sex, and TCR diversity (D50)

## Introduction

Lung cancer is the deadliest malignancy in the world. According to GLOBOCAN 2022, lung cancer was the most frequently diagnosed cancer and the primary cause of cancer-related deaths worldwide in 2022, accounting for an estimated 2.5 million new cases and 1.8 million deaths globally.^1^ Clinically, early-stage lung cancer was often challenging to detect based on symptoms, leading to the majority of patients being diagnosed at advanced or even metastatic stage, with a five-year survival rate of approximately 15%,^2^ highlighting the importance of early diagnosis. The gold standard for diagnosis of lung cancer is pathological biopsy, with an accuracy of approximately 87%.^3^^,^^4^ Though a number of antigens found in blood have been assessed as potential biomarkers of lung cancer,^5^^,^^6^ a single antigen biomarker lacked sufficient diagnostic value. In recent years, low-dose computed tomography (LDCT) has been established as the standard screening method for lung cancer and reduces mortality among high-risk individuals.^3^ However, LDCT identifies pulmonary nodules in approximately 20% of screened subjects, while its 96.4% false-positive rate leads to excessive medical care and psychological distress.^7^ Additionally, CT scans pose risks due to cumulative radiation exposure,^8^ particularly in relation to potential increases risks of brain cancers, leukemia, and many other types of cancers.^9^^,^^10^^,^^11^ Thus, an accurate and non-invasive diagnostic method is critically needed for early-stage lung cancer prediction.

Liquid biopsy is a non-invasive screening technique,^12^ and the screening results are quantifiable, objective, and free from unnecessary radiation exposure compared with LCDT. However, the sensitivity was low for detection of stage I lung cancer with the sensitivity of <10%.^13^ This may be the result of the majority of mutations identified in plasma cell-free DNA (cfDNA) originate from white blood cells rather than from cancer cells.^14^ During cancer progression, the tumor-infiltrating T cells recognize tumor-specific antigens, leading to the clonal expansion of T cells with unique TCR.^15^ These tumor-reactive T cells are detectable in peripheral blood. TCR sequencing has shown great potential in disease diagnosis.^16^^,^^17^ Prior studies in lung cancer have demonstrated both the feasibility of blood TCR repertoire-based detection and insights into T cell response heterogeneity within tumors.^18^^,^^19^ However, few studies focused on its applications for early-stage lung cancer prediction by using TCR-seq data. In previous work, we developed TCRnodseek, a TCR-based model utilizing three key features (ground glass nodule, Shannon index, and evenness index), to discriminate malignant from benign lung nodules, achieving an AUC of 0.80 in the validation cohort.^20^ Building upon this foundation, we subsequently established the LungTCR database (https://www.lungtcr.com/) through large-scale TCR sequencing (6,059 blood and 988 tumor samples) and developed TCRnodseek plus, an enhanced model integrating clinical, CT imaging, and TCR features, which demonstrated superior diagnostic performance in a multi-center prospective study.^21^ Given the risks associated with CT imaging, we established a CT-free method for early lung cancer diagnosis. In the validation cohort, this method archived moderate discriminative performance (AUC: 0.72).^22^ Collectively, these findings demonstrate the significant diagnostic potential of TCR-based profiling for lung cancer detection.

In this study, we present LCPBert, a deep learning framework for non-invasive early-stage lung cancer detection leveraging TCR repertoires. By incorporating lung cancer risk index (LCRI), LCPBert provided a quantitative measure of individual lung cancer risk. Our study highlighted LCPBert as a non-invasive, accurate, and dynamic diagnostic tool.

## Results

### Early-stage lung cancer patients demonstrate reduced TCRβ diversity and higher clonalities compared with healthy donors

To investigate T cell immune responses, TCRβ repertoires in peripheral blood were profiled via high-throughput sequencing in cohorts of healthy donors and early-stage lung cancer patients. In total, we obtained almost 1.5 × 10^8^ TCRβ sequences from 2,699 healthy donors and 3,360 early-stage lung cancer patients (Figure S1).

**Figure 1:**
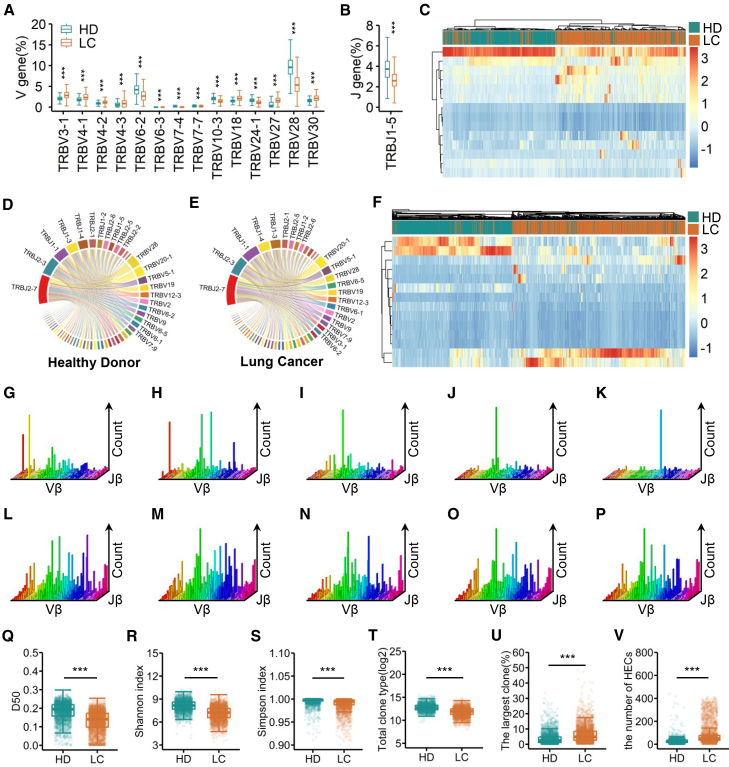
Early-stage lung cancer patients demonstrate reduced TCRβ diversity and higher clonalities compared with healthy donors The usage frequencies of (A) 14 significantly different TRBV and (B) 1 significantly different TRBJ genes in the early-stage lung cancer patients (LC, *n* = 2,699) and the healthy donors (HD, *n* = 3,360). The error bars indicated standard deviations. (C) The heatmap of expansion frequencies of the 14 significantly different TRBV genes in the LC and the HD. TRBV and TRBJ combination in the TCR repertories of (D) the LC and (E) the HD. (F) The heatmap of expansion frequencies of the 195 significantly different V-J pairs in the LC and the HD. (G–P) The V-J combinations were analyzed using the 3D plots, G to K, representative examples of the LC; (L–P) representative examples of the HD. *x* and *y* axes depict functional human TRBV and TRBJ alleles, respectively. *z* axis indicates the counts of sequence reads. Comparison of (Q) D50, (R) Shannon index, (S) Simpson index, (T) total clone type, (U) the frequencies of the largest clone, and (V) the number of HECs in the HD versus the LC. Data are represented as mean ± SD. ∗, *p* < 0.05, ∗∗, *p* < 0.01, ∗∗∗, *p* < 0.001, Mann-Whitney U test.

In our study, among the 47 functional human TRBV and the 13 functional human TRBJ genes, 14 distinct Vβ and 1 distinct Jβ genes were identified (Table S1). We found that the usage frequencies of 7 distinct Vβ genes (TRBV3-1, TRBV4-1, TRBV4-2, TRBV4-3, TRBV18, TRBV27, and TRBV30) were significantly elevated in the LC compared with those in the HD; in contrast, the usage frequencies of seven distinct Vβ genes (TRBV6-2, TRBV6-3, TRBV7-4, TRBV7-7, TRBV10-3, TRBV24-1, and TRBV28) were significantly reduced in the LC compared with those in the HD (Figure 1A, *p* < 0.05). In addition, the usage frequency of 1 distinct Jβ gene (TRBJ1-5) was significantly reduced in the LC compared with that in the HD (Figure 1B, *p* < 0.05). Furthermore, the majority of LC were clustered together in the expansion of 14 distinct Vβ genes (Figure 1C).

There were also obvious differences in V-J combinations between repertoires of the early-stage lung cancer patients and the healthy individuals (Figures 1D and 1E). We compared the usage frequencies of 611 V-J pairs in the LC and the HD, and found that 195 V-J pairs exhibited significant differences. Among them, 75 pairs showed significantly increased frequencies in the LC compared with the HD, while the frequencies of the remaining 120 pairs were significantly decreased in the LC (Table S2). Most lung cancer samples were clustered within the expansion of 195 distinct V-J pairs (Figure 1F). The 3-D V-J recombination plots revealed markedly skewed distributions in both sample types, characterized by dominant single-column patterns indicative of TCR repertoire imbalance. Notably, this skewness was significantly more pronounced in the lung cancer samples (Figures 1G–1K) than in the healthy individuals (Figures 1L–1P).

we calculated D50, Shannon index, Simpson index, total clone type, the frequency of the largest clone, and the number of HECs in each peripheral blood sample. Significant differences were found between the early-stage lung cancer patients and the healthy samples. The results showed that D50, Shannon index, Simpson index, total clone type in the lung cancer samples (mean ± standard deviation [SD]; D50: 0.1318 ± 0.0548; Shannon indexes: 7.1284 ± 0.9132; Simpson indexes: 0.9892 ± 0.0164; total clone type: 4,458.864 ± 2,838.531) were significantly lower than those in the benign ones (D50: 0.1844 ± 0.0562, *p* < 0.001; Figure 1Q; Shannon indexes: 8.1210 ± 0.7359, *p* < 0.001; Figure 1R; Simpson indexes: 0.9956 ± 0.0089, *p* < 0.001; Figure 1S; total clone type: 8,002 ± 4,643.461, *p* < 0.001; Figure 1T). TCRβ genes obtained from the LC (6.38% ± 5.31%) showed significantly higher frequencies of the largest TCR clone compared with the HD (3.70% ± 3.87%, *p* < 0.001; Figure 1U). Additionally, greater number of HECs were observed in the LC (83.5482 ± 85.6583) than those in the HD (28 ± 25.9233, *p* < 0.001; Figure 1V). These results suggested that early-stage lung cancer patients demonstrate reduced TCRβ diversity and increased clonality compared with healthy donors.

### Development of the LCPBert framework

**Figure 2:**
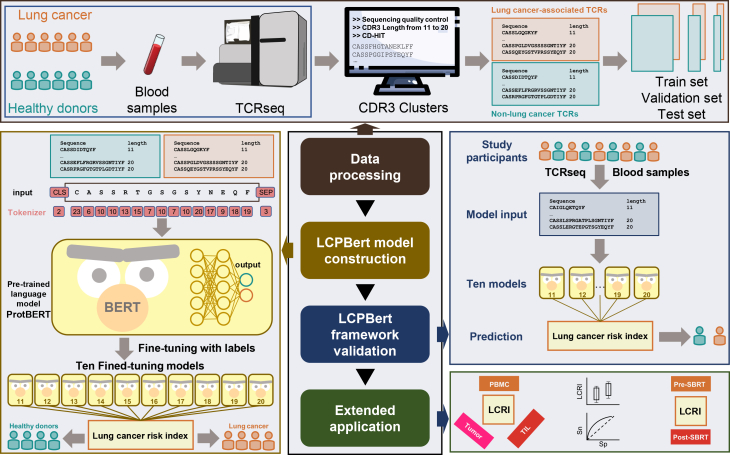
Development of the LCPBert framework The framework is divided into three parts: data processing, LCPBert model construction, and LCPBert framework validation. (1) Data processing: non-lung cancer TCRs were obtained from peripheral blood TCR-seq samples of the healthy donors (HD, *n* = 2,199). Lung cancer-associated TCRs were derived from peripheral blood TCR-seq samples of the early-stage lung cancer patients (LC, *n* = 2,860). Lung cancer-associated TCRs that were shared with non-lung cancer TCRs were excluded. (2) LCPBert model construction: CDR3 sequences representing ten discrete length variants (11–20 amino acids) were tokenized and processed through the pre-trained ProtBERT model. (3) LCPBert framework validation: lung cancer risk assessment was quantified using LCRI. CDR3 sequences of lengths 11–20 amino acids were subsequently processed through their corresponding length-specific LCPBert models to compute individual LCRI values, which served as diagnostic classifiers for lung cancer status.

In this study, we developed a ProtBERT-based framework named LCPBert for lung cancer prediction utilizing T cell receptors from peripheral blood. The framework is divided into three parts: data processing, LCPBert model construction, and LCPBert framework validation (Figure 2).

In the data processing phase, peripheral blood TCR repertoire analysis yielded 300,140 non-lung cancer CDR3 sequences from 2,199 healthy donors and 261,579 lung cancer-associated CDR3 sequences from 2,860 patients. To ensure tumor specificity, all CDR3 sequences shared with healthy donors were systematically excluded prior to downstream analysis. CDR3 sequences were stratified by length (11–20 amino acids) and randomly sampled to construct the test set, with 500 sequences selected per length stratum (total *n* = 10,000). The remaining sequences were partitioned into training and validation sets at a 4:1 ratio via randomized allocation (Table S3).

In the LCPBert model construction phase, CDR3 sequences representing ten discrete length variants (11–20 amino acids) were tokenized and processed through the pre-trained ProtBERT model. For each length variant, an independent fine-tuning procedure was implemented with task-specific classification heads, optimizing hyperparameters including learning rate adjustments. This generated ten length-specific LCPBert models for downstream analysis.

In the LCPBert framework validation phase, lung cancer risk assessment was quantified using LCRI. Peripheral blood-derived TCR CDR3 sequences from study participants underwent high-throughput sequencing (TCR-seq). CDR3 sequences of lengths 11–20 amino acids were subsequently processed through their corresponding length-specific LCPBert models to compute individual LCRI, which served as diagnostic classifiers for lung cancer status.

### LCPBert demonstrated good performance on predicting lung cancer-associated TCRs

In this study, we identified a total of 15,038,284 unique TCR CDR3 sequences, consisting of 8,275,774 sequences from 3,360 healthy individuals and 8,420,220 sequences from 2,699 early-stage lung cancer patients with 1,657,710 sequences shared between cohorts (Figure S1). CDR3 length profiling demonstrated that 11–20 amino acid sequences constitute 99.28% of lung cancer and 99.29% of non-lung cancer repertoires (Figure S2).

**Figure 3:**
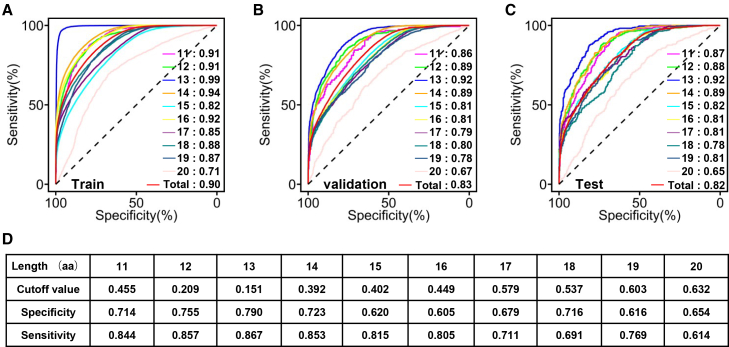
LCPBert demonstrated good performance on predicting lung cancer-associated TCRs ROC curves showing the performance of LCPBert to distinguish lung cancer-associated TCRs from non-lung cancer TCRs across (A) the train set, (B) the validation set, and (C) the test set. Different models were applied to CDR3 sequences of varying lengths, ranging from 11 to 20 amino acids. Different models were applied to CDR3 sequences ranging from 11 to 20 amino acids in length, and their diagnostic performance for lung cancer-associated TCRs varied. (D) The optimal cutoff values and the corresponding specificity and sensitivity for models trained on different CDR3 lengths in the validation set.

LCPBert models trained with CDR3 sequences of 11–20 amino acids lengths achieved training AUCs of 0.91 (11aa), 0.91 (12aa), 0.99 (13aa), 0.94 (14aa), 0.82 (15aa), 0.92 (16aa), 0.85 (17aa), 0.88 (18aa), 0.87 (19aa), and 0.71 (20aa) (overall AUC = 0.90; Figure 3A). Validation performance yielded 0.86 (11aa), 0.89 (12aa), 0.92 (13aa), 0.89 (14aa), 0.81 (15aa), 0.81 (16aa), 0.79 (17aa), 0.80 (18aa), 0.78 (19aa), and 0.67 (20aa) (overall AUC = 0.83; Figure 3B). Test set evaluation showed 0.87 (11aa), 0.88 (12aa), 0.92 (13aa), 0.89 (14aa), 0.82 (15aa), 0.81 (16aa), 0.81 (17aa), 0.78 (18aa), 0.81 (19aa), and 0.65 (20aa) (overall AUC = 0.82; Figure 3C). LCPBert consistently discriminated lung cancer-associated from non-lung cancer CDR3 sequences across nearly all length variants. Furthermore, length-specific optimal probability thresholds were determined from the validation set to classify a TCR clone as lung cancer-associated for subsequent LCRI calculation: 0.455 (11aa), 0.209 (12aa), 0.151 (13aa), 0.392 (14aa), 0.402 (15aa), 0.449 (16aa), 0.579 (17aa), 0.537 (18aa), 0.603 (19aa), and 0.632 (20aa; Figure 3D).

### LCRI as a robust predictor for early-stage lung cancer

**Figure 4:**
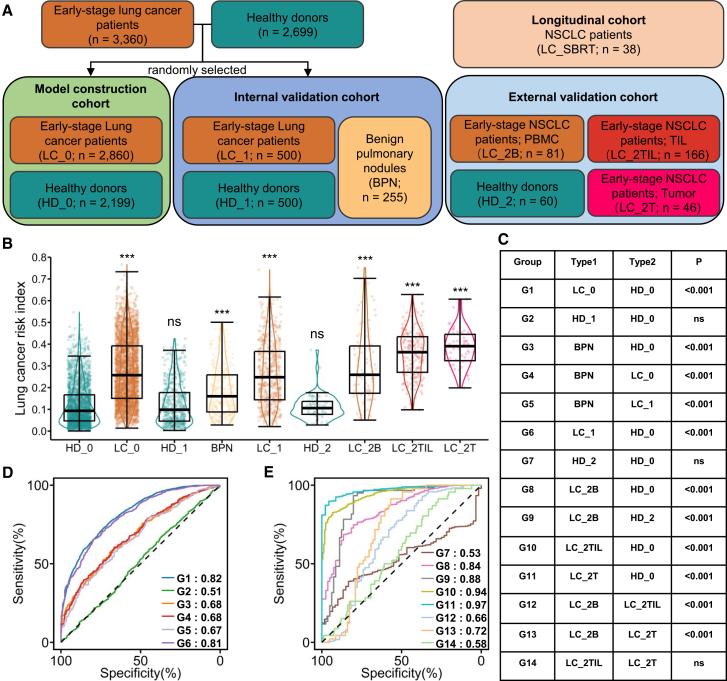
LCRI as a robust predictor for early-stage lung cancer (A) Sample division in this study. (B) The LCRI distributions for four cohorts: (1) model construction cohort: HD_0 (healthy donors, *n* = 2,199), LC_0 (early-stage lung cancer patients, *n* = 2,860), (2) internal validation cohort: HD_1 (healthy donors, *n* = 500), BPNs (benign pulmonary nodules, *n* = 255), LC_1 (early-stage lung cancer patients, *n* = 500), (3) external validation cohort: HD_2 (healthy donors, *n* = 60), LC_2B (peripheral blood from NSCLC patients, *n* = 81), LC_2TIL (tumor-infiltrating lymphocytes from NSCLC patients, *n* = 166), LC_2T (tumor tissue from NSCLC patients, *n* = 46). Data are represented as mean ± SD. ∗, *p* < 0.05, ∗∗, *p* < 0.01, ∗∗∗, *p* < 0.001, Mann-Whitney U test. (C) *p* values for the twelve comparison groups, G1 represents model construction cohort, G2-G5 represent the internal validation cohort, G6-G12 represent the external validation cohort. (D and E) ROC curves of twelve comparison groups.

Building upon the discriminative capacity of LCPBert for lung cancer-associated CDR3 sequences, we quantified individualized cancer risk using the LCRI across 6,705 TCR-seq samples categorized into three cohorts: (1) model construction cohort, including HD_0 (healthy donors, *n* = 2,199) and LC_0 (early-stage lung cancer patients, *n* = 2,860); (2) internal validation cohort, including HD_1 (healthy donors, *n* = 500), BPN (benign pulmonary nodules, *n* = 255), and LC_1 (early-stage lung cancer patients, *n* = 500); and (3) external validation cohort, including HD_2 (healthy donors, *n* = 60), LC_2B (peripheral blood from NSCLC patients, *n* = 81), LC_2TIL (tumor-infiltrating lymphocytes from NSCLC patients, *n* = 166), and LC_2T (tumor tissue from NSCLC patients, *n* = 46) (Figure 4A; Table 1).

**Table 1:** Concise summary for the study cohorts

Group	Disease	Cohort	Sample Size	Age (Median [IQR, Range])
HD_0	HD	Model construction cohort	2,199	40.0 [33.0–50.0, 5–87]
LC_0	LC	Model construction cohort	2,860	62.0 [54.0–69.0, 18–94]
HD_1	HD	Internal validation cohort	500	43.0 [33.0–52.0, 22–83]
BPN	BPN	Internal validation cohort	255	52.0 [46.0–58.0, 26–81]
LC_1	LC	Internal validation cohort	500	63.0 [54.0–70.0, 23–92]
HD_2	HD	External validation cohort	60	N/A^a^
LC_2B	NSCLC	External validation cohort	81	N/A^a^
LC_2TIL	NSCLC	External validation cohort	166	N/A^a^
LC_2T	NSCLC	External validation cohort	46	N/A^a^
LC_SBRT	NSCLC	Longitudinal cohort	38	N/A^a^

a age information was not available from the original public databases.

In the model construction cohort, the LCRI was significantly higher in LC_0 (mean ± SD: 0.275 ± 0.146) than in HD_0 (0.120 ± 0.093; *p* < 0.001). In the internal validation cohort, LCRI values were significantly elevated in LC_1 (0.263 ± 0.142, *p* < 0.001 vs. HD_0) and BPN (0.184 ± 0.113, *p* < 0.001 vs. HD_0), while HD_1 showed no significant difference from HD_0 (0.124 ± 0.095, *p* > 0.05). Comparisons among these groups revealed that LC_1 had significantly higher LCRI than BPN (*p* < 0.001), and BPN had significantly higher LCRI than HD_1 (*p* < 0.001; Figures 4B and 4C). These results demonstrated that LCRI values were highest in LC patients, followed by BPN patients, and lowest in HD. This stepwise increase across clinical states confirmed robust generalization of LCRI across distinct clinical subgroups.

To further validate generalizability of LCRI, we analyzed an external validation cohort comprising NSCLC samples from three independent studies. The LCRI in HD_2 (0.111 ± 0.058, *p* > 0.05) remained comparable to HD_0. Conversely, all cancer-derived samples exhibited significantly higher LCRI: (1) LC_2B (peripheral blood): 0.296 ± 0.166; (2) LC_2TIL (tumor-infiltrating lymphocytes): 0.355 ± 0.105; and (3) LC_2T (tumor tissue): 0.384 ± 0.084 (all *p* < 0.001 vs. HD_0) (Figures 4B and 4C).

Notably, the LCRI values increased progressively from peripheral blood samples (LC_2B), TILs samples (LC_2TIL), to tumor tissue samples (LC_2T) (*p* < 0.001 for LC_2B vs. LC_2TIL; *p* < 0.001 for LC_2B vs. LC_2T). This gradient underscores the capacity of LCPBert to discriminate cancer signals across biological compartments and further validates its superior generalizability in diverse sample types (Figures 4B and 4C).

Discriminative performance was quantified via AUC for twelve comparative groups (G1–G14): G1–G6 (model construction/internal validation cohort): 0.82, 0.51, 0.68, 0.68, 0.67, and 0.81 (Figure 4D); G7–G14 (external validation cohort): 0.53, 0.84, 0.88, 0.94, 0.97, 0.66, 0.72, and 0.58 (Figure 4E). Based on ROC curve analysis of G1 group (model construction cohort), we established an LCRI cutoff value of 0.1465, which achieved a sensitivity of 0.75 and specificity of 0.70 in discriminating lung cancer patients from healthy individuals. When applied to the external validation cohort G9 (LC_2B vs. HD_2), this same cutoff maintained a high diagnostic performance, achieving a sensitivity of 0.78 and a specificity of 0.83.

### Elevated LCRI predicted distant metastasis in NSCLC patients

Radiotherapy is recognized to exert profound immunomodulatory effects, with its capacity to recruit antigen-specific T cells emerging as a pivotal mechanism of interest. Here, we collected a longitudinal cohort (*n* = 19 patients) undergoing paired LCRI measurements at 24 h before and after stereotactic body radiotherapy (SBRT),^23^ integrating longitudinal distant metastasis (DM) status to reveal that LCRI trajectories stratify distinct immunophenotypic responses.

**Figure 5:**
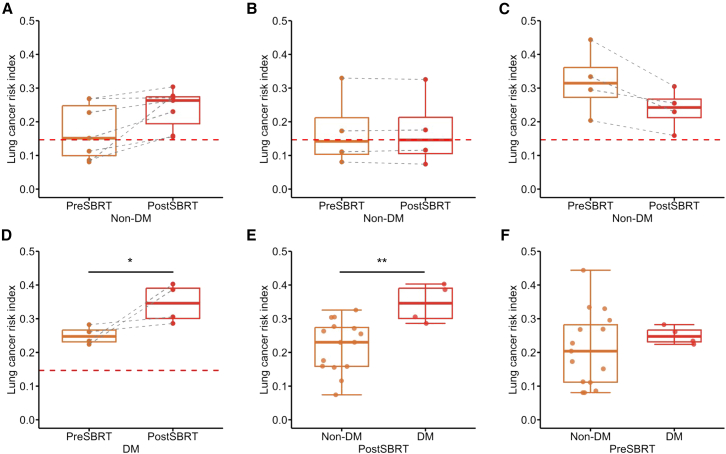
Elevated LCRI predicted distant metastasis in NSCLC patients (A–D) Changes in LCRI and treatment outcomes after SBRT. Boxplots showing differences in LCRI in baseline (before-SBRT) samples; (A) positive induction subtype in non-DM patients, (B) temporal stability subtype in non-DM patients, (C) reduction subtype in non-DM patients, and (D) DM patients. Boxplots showing differences in (E) post-SBRT LCRI and (F) pre-SBRT LCRI between non-DM patients and DM patients. The cutoff values are indicated with dashed red lines.

Analysis of the 15 non-DM patients revealed three distinct LCRI response patterns based on a predefined threshold of meaningful change, specifically |ΔLCRI| > 0.01. (1) A positive induction subtype (40.0%, 6/15) displayed a clear post-SBRT increase in LCRI, for example in P03 (0.15–0.23; Figure 5A; Table S4). (2) A temporal stability subtype (33.3%, 5/15) showed minimal fluctuation (|ΔLCRI| ≤ 0.01), as seen in P07 (0.08–0.07; Figure 5B; Table S4). (3) A reduction subtype (26.7%, 4/15) demonstrated a decrease in LCRI, illustrated by P01 (0.33–0.23; Figure 5C; Table S4).

Critically, all DM patients (*n* = 4) exhibited a significant increase in LCRI post-SBRT (*p* < 0.05; Figure 5D; Table S4). Within this group, the response was pronounced. Half (50%, 2/4) of DM patients showed a substantial LCRI elevation greater than 0.15, and most (75%, 3/4) maintained a high post-treatment LCRI above 0.30. As a result, post-SBRT LCRI was significantly higher in the DM cohort than in the non-DM cohort (*p* < 0.01; Figure 5E; Table S4), a difference not observed pre-SBRT (Figure 5F; Table S4). These findings suggested that a robust post-radiotherapy expansion of the putative tumor-reactive TCR repertoire is associated with the metastatic phenotype.

### LCRI predicted lung cancer independently of age, sex, and TCR diversity (D50)

To evaluate the influence of age, sex, and D50 on the predictive performance of LCRI, we conducted a comprehensive analysis within the internal validation cohort (*n* = 1,248).

**Figure 6:**
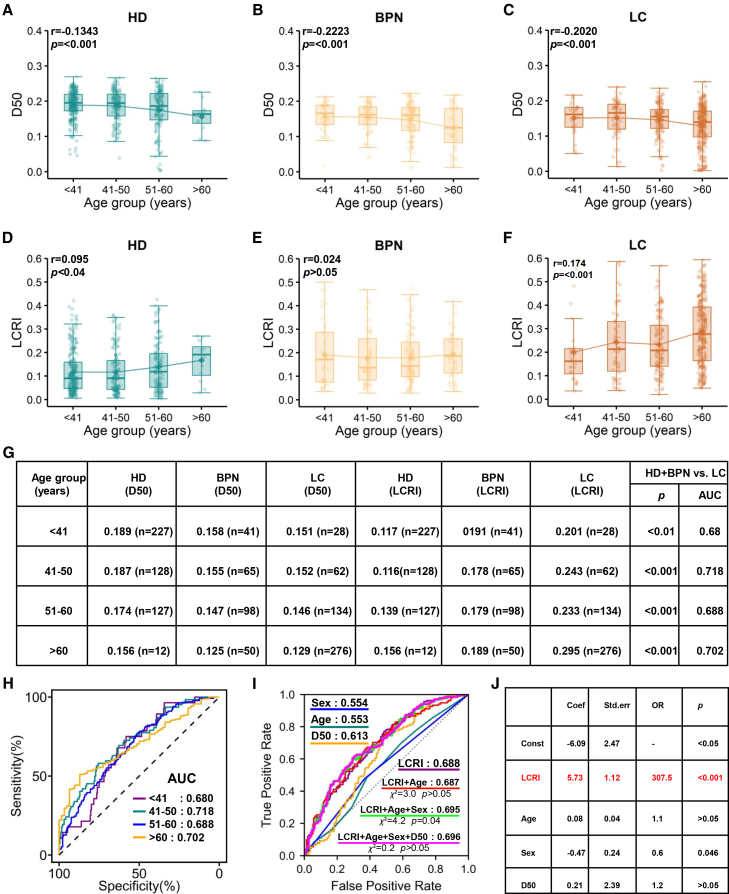
LCRI predicted lung cancer independent of age, sex, and TCR diversity (D50) Correlation analysis between age and TCR repertoire diversity (D50) in (A) healthy donors, (B) benign pulmonary nodule patients, and (C) lung cancer patients. Correlation analysis between age and LCRI in (D) healthy donors, (E) benign pulmonary nodule patients, and (F) lung cancer patients. (G) Comparison of TCR diversity (D50) and LCRI across age-stratified groups, illustrating the hierarchy among healthy donors, benign pulmonary nodule patients, and lung cancer patients. (H) ROC curves for LCRI distinguishing lung cancer patients from controls (HD + BPN) across four age groups. (I) Comparison of AUCs for the LCRI model alone, models using individual variables (age, sex, and D50), and the stepwise addition of these variables to the LCRI model. (J) Multivariable logistic regression analysis showing the independent contributions of LCRI, age, sex, and D50 after mutual adjustment.

We first validated the relationship between age and TCR diversity (D50) in our cohort, observing a significant negative correlation. Previous research demonstrated that TCR repertoire diversity progressively declines with age in both healthy individuals and cancer patients, primarily due to reduced clonal richness.^24^ We confirmed this negative correlation in HD (r = −0.1343, *p* < 0.001; Figure 6A), BPN patients (r = −0.2223, *p* < 0.001; Figure 6B), and LC patients (r = −0.2020, *p* < 0.001; Figure 6C). Stratified analysis revealed a consistent hierarchy where healthy donors maintained the highest TCR diversity at all ages, followed by BPN patients, while LC patients exhibited the lowest levels.

We performed multiple linear regression analysis modeling D50 as a function of age and lung cancer status. As expected, age was a significant negative predictor of D50 (β = −0.00106, *p* < 0.001). After adjusting for age, lung cancer status remained a highly significant independent predictor of D50 (β = −0.01724, *p* < 0.001, Table S5).

We next examined the relationship between age and LCRI. Weak but significant positive correlations were observed in HD (r = 0.095, *p* < 0.05; Figure 6D) and LC patients (r = 0.17, *p* < 0.05; Figure 6F), whereas no significant correlation was found in BPN patients (r = 0.024, *p* > 0.05; Figure 6E). Notably, the sample distribution across age groups was imbalanced in healthy donors and LC patients, which may partially account for these weak correlations. Stratified analysis demonstrated that LC patients consistently exhibited the highest LCRI values across all age groups, followed by BPN patients, with HD showing the lowest levels (Figure 6D). Furthermore, LCRI effectively distinguished LC patients from controls (HD + BPN) across all four age groups (<41, 41–50, 51–60, and >60 years), with AUCs of 0.68, 0.718, 0.688, and 0.702, respectively (all *p* < 0.01; Figures 6G and 6H).

To further assess the relative contributions of age, sex, and D50 to the predictive performance of LCRI, we performed stepwise multivariable logistic regression analysis in the 51–60 years subgroup, which had a relatively balanced sample distribution (controls, HD + BPN: *n* = 225; LC: *n* = 134). Models incorporating only sex, only age, or only D50 yielded AUCs of 0.554, 0.553, and 0.613, respectively, indicating limited diagnostic value for these factors alone within this age stratum. The model incorporating only LCRI achieved an AUC of 0.688 (Figure 6I). Sequential addition of variables revealed incremental contributions. Adding age marginally decreased the AUC to 0.687 (ΔAUC = −0.001; likelihood ratio test *p* > 0.05; Figure 6I). Subsequent addition of sex increased the AUC to 0.695 (ΔAUC = +0.008; likelihood ratio test *p* = 0.04; Figure 6I). Adding D50 resulted in a negligible AUC improvement to 0.696 (ΔAUC = +0.001; likelihood ratio test *p* > 0.05; Figure 6I). In the full multivariable model, age and D50 lost their independent significance (both *p* > 0.05; Figure 6J), while sex remained statistically significant (*p* < 0.05) but contributed only a minimal improvement in model performance. Notably, LCRI maintained robust independent predictive power after adjusting for age, sex, and D50 (OR = 307.5, *p* < 0.001; Figure 6J).

Collectively, these results demonstrated that LCRI not only distinguished LC patients from controls across all age groups but also captured lung cancer-associated immune signals that are independent of chronological aging, as confirmed within a specific age stratum.

## Discussion

In this study, we analyzed peripheral TCR repertoires to characterize T cell immune responses differences between the healthy individuals and early-stage lung cancer patients. Our results revealed that lung cancer patients exhibited reduced diversity indices (D50, Shannon index, Simpson index; *p* < 0.001) and increased antigen-driven clonal expansion (elevated HECs and the biggest clone frequency) compared with healthy controls. The observed differential Vβ/Jβ gene usage (14 genes altered) and skewed V-J pair distribution (195 differentially expressed pairs) reflected antigen-driven remodeling of the TCR repertoire. Notably, some of these reduced genes, particularly TRBV6-2, were associated with MAIT cells, which are known to decline with age.^25^^,^^26^^,^^27^ In addition, the lung cancer cohort was significantly older than the control cohort, and therefore these observed differences may be partially confounded by age and sample imbalances. To address this, stratified analysis revealed a consistent hierarchy where healthy donors maintained the highest TCR diversity at all ages, followed by BPN patients, while LC patients exhibited the lowest levels, indicating that this diversity loss is not merely a reflection of age but is specifically associated with malignancy. These perturbations align with the tumor immunoediting paradigm, wherein malignant cells evade immunosurveillance by promoting oligoclonal T cell exhaustion while suppressing broad antigen recognition capacity.^28^^,^^29^

As part of the TCR variable chain, CDR3 is crucial for T cell recognition of antigens, and it directly reflects the T cell immune response status. Building on these findings, we developed LCPBert, a deep learning framework for lung cancer prediction based on peripheral blood TCRβ-chain sequences. By fine-tuning ProtBERT, a bidirectional transformer-based protein language model that generates contextual residue embeddings, LCPBert effectively distinguished lung cancer-associated TCRs from non-lung cancer TCRs in test set (AUC = 0.82).

The adaptive immune system is very important in fighting diseases, especially cancer. T cells are an important weapon of the body’s anti-tumor immune response, and they specifically recognize tumor cells through their TCR and thereby selectively remove them. Most of the tumor-associated T cells that recognize and kill cancer cells are eliminated from the circulation along with the effective clearance of precancerous and/or cancerous cells.^30^ During tumorigenesis, the TCR repertoire would under constant turnover driven by the emergence and clearance of sporadic cancer cells. However, upon progression to the “equilibrium” or “escape” states of cancer immunoediting,^31^ tumor-associated T cells exhibit sustained generation and progressive accumulation due to exhaustion rather than apoptosis.^32^ Thus, the accumulated tumor-specific TCR repertoire features can be detected and reflect tumor cell presence. Consistent with this premise, cancer-associated T cells were detectable in peripheral blood from patients with early-stage colorectal cancer, melanoma, and lung cancer.^18^^,^^19^^,^^33^^,^^34^ Consider about this, we introduced the LCRI as a potential biomarker aggregating expression frequencies of lung cancer-associated TCRs to quantify individual cancer risk. This mechanistic link confirms that LCRI was biologically underpinned by antigen-driven immune responses, reflecting its immunological validity for clinical stratification.

The LCRI, developed by the LCPBert framework, quantifies T cell receptor dysregulation with high clinical utility. In the internal validation cohorts, LCRI values demonstrated a progressive increase from healthy donors (mean = 0.12) to patients with benign pulmonary nodules (0.18), and ultimately to lung cancer patients (0.28), mirroring neoplastic progression dynamics. This finding was reinforced by the spatial gradient of LCRI across anatomical compartments in the external validation cohorts: LCRI values progressively increase from peripheral blood (0.28) to tumor-infiltrating lymphocytes (TILs, 0.34) and peak in tumor tissue (0.37), reflecting compartment-specific tumor burden and localized immune evasion within the tumor microenvironment. Traditional serum markers often lack sufficient sensitivity or specificity for early-stage detection.^35^ In contrast, the LCRI proposed in this study demonstrates significantly superior diagnostic performance. Previous studies reported that carcinoembryonic antigen (CEA) had a sensitivity of 69% and specificity of 68% for lung cancer diagnosis, whereas cytokeratin 19 fragment (CYFRA 21-1) showed 43% sensitivity and 89% specificity.^5^ In the external validation cohort (HD_2 vs. LC_2B), LCRI was adjusted to match these specificity levels (68% for CEA, 89% for CYFRA 21-1), achieving sensitivities of 97% and 67%, respectively. This indicated that, under equivalent specificity conditions, LCRI’s sensitivity was significantly higher than both CEA (97% vs. 69%) and CYFRA 21-1 (67% vs. 43%).

We acknowledge that due to age imbalances in the training and validation datasets, some of the signal captured by LCRI may reflect age-associated immune signatures. In the external validation comparison between HD_2 and LC_2B, if the age distribution is substantially different between these two groups, this imbalance could contribute to the observed discriminative performance. However, our internal validation analyses demonstrate that LCRI consistently distinguishes lung cancer patients from controls across all age groups in age-stratified analysis. In the 51–60 years subgroup where age was balanced between cases and controls, LCRI maintained consistent predictive performance (AUC = 0.688) after adjusting for age, sex, and D50, while age alone showed no discriminative ability. These findings indicate that LCRI reflects lung cancer-associated immune signals rather than general age-related changes in the TCR repertoire. Thus, although age may play a confounding role in some external comparisons, the core signal captured by LCRI is fundamentally independent of chronological aging.

Despite its widespread adoption in lung cancer screening, LDCT is associated with high false-positive rates. In addition, LDCT exposed patients to ionizing radiation. If current CT practices persisted, the scans could eventually account for an estimated 5% of new cancer diagnoses each year.^11^ Liquid biopsy approaches avoid radiation exposure compared with LDCT. However, limitation of liquid biopsies was showed in early-stage lung cancer prediction with lower sensitivity.^13^ In this study, based on LCRI, LCPBert exhibited dual prognostic utility. As a screening adjunct, it achieves 75% sensitivity and 70% specificity at the established cutoff (0.1465), while eliminating radiation exposure risk. As a dynamic monitor, In DM patients, a substantial LCRI elevation greater than 0.15 was observed in 50% (2/4) of cases and 75% (3/4) maintained a high post-treatment LCRI above 0.30. Thus, this underscores the potential of LCPBert as a safer and more efficient tool for lung cancer screening and monitoring, which could potentially enhance early detection and improve patient outcomes.

In conclusion, we developed LCPBert, a deep learning framework for early-stage lung cancer prediction, leveraging TCR repertoire analysis to enable non-invasive screening. The LCRI-based approach demonstrated strong diagnostic performance in identifying early-stage patients and showed prognostic potential for guiding immunotherapeutic strategies in post-SBRT cohorts. Furthermore, LCRI predicted lung cancer independent of age, sex, and TCR diversity (D50). These findings collectively highlight the capacity of TCR-based biomarkers to overcome limitations of traditional serum markers. LCPBert’s non-invasive design, requiring only peripheral blood, also enables repeatable clinical deployment compared with conventional diagnostic methods.

### Limitations of the study

This study has several limitations. First, a significant age mismatch exists between our retrospectively collected cohorts, including the external validation cohort. This age disparity represents a key confounding factor. Future prospective studies with better matching for age are necessary to validate our findings reliably. Second, the performance of LCPBert was not assessed across distinct histological subtypes of lung cancer due to limited cohort sizes. Expanding and balancing these cohorts is required for a more nuanced evaluation. Third, our current model is based solely on β-chain sequencing data. The predictive capacity may be enhanced by incorporating paired αβ-chain sequencing data in future work.

## Resource availability

### Lead contact

Requests for further information and resources should be directed to and will be fulfilled by the lead contact, Jian Huang (hj@uestc.edu.cn).

### Materials availability

This study did not generate new unique reagents.

### Data and code availability

• This study analyzes existing publicly available data and those datasets are listed in the key resources table. The data are publicly available as of the date of publication.
• The code of LCPBert is available on GitHub in the repository LCPBert_Project (https://github.com/xinguoguo9211/LCPBert_Project). The code is publicly available as of the date of publication.
• The project uses ProtBERT (Rostlab/prot_bert) as the base model and must be downloaded prior to training from HuggingFace Model Hub (https://huggingface.co/Rostlab/prot_bert).
• Any other additional information required to reanalyze the data reported in this study is available from the lead contact upon request.

## Acknowledgments

We thank all the patients who participated in this study. This study was supported by Medico-Engineering Cooperation Funds from 10.13039/501100005408University of Electronic Science and Technology of China (ZYGX2022YGRH004), the 10.13039/501100001809National Natural Science Foundation of China (62371112), and the Incubation Program for Innovative Science and Technology in UESTC (award no. Y03023206100209).

## Author contributions

Conceptualization, J.H., H.L., and X.Y.; methodology, X.Y., Y.Z., and Z.Z.; investigation, H.L.; writing – original draft, X.Y.; funding acquisition, J.H. and H.L.; resources, J.H. and H.L.; supervision, J.H. and H.L.

## Declaration of interests

The authors declare no competing interests.

## STAR★Methods

### Key resources table

**Table:** 

REAGENT or RESOURCE	SOURCE	IDENTIFIER
**Deposited data**		

3,360 peripheral blood samples from lung cancer patients	This paper	GSA: HRA008198
2,699 peripheral blood samples from healthy individuals	This paper	GSA: HRA007853
255 peripheral blood samples from patients with benign pulmonary nodules	This paper	GSA: HRA006703
42 peripheral blood samples from healthy individuals	Warren RL et al.^36^ AND TCRdb2.0	SRA: PRJNA79707 AND https://guolab.wchscu.cn/TCRdb2/#/display/project/PRJNA79707
18 peripheral blood samples from healthy individuals	NCBI SRA BioProject AND TCRdb2.0	SRA: PRJNA258001 AND https://guolab.wchscu.cn/TCRdb2/#/display/project/PRJNA258001
81 peripheral blood samples, 166 tumor-infiltrating lymphocyte (TIL) samples and 46 tumor tissue samples from patients with early-stage non-small cell lung cancer (NSCLC)	Kroopa Joshi et al.^37^ AND TCRdb2.0	SRA: PRJNA544699 AND https://guolab.wchscu.cn/TCRdb2/#/display/project/PRJNA544699
38 peripheral blood samples from 19 NSCLC patients, collected at matched time points both pre- and 24 hours post-stereotactic body radiation therapy (SBRT)	Wu, L. et al.^23^ AND TCRdb2.0	SRA: PRJNA767497 AND https://guolab.wchscu.cn/TCRdb2/#/display/project/PRJNA767497

**Software and algorithms**		

Python (version 3.10.14)	Python Software Foundation	https://www.python.org/
PyTorch (version 1.2.1)	PyTorch software	https://pytorch.org/
R (version 4.2.3)	R software	https://www.r-project.org
ProtBERT	Rostlab	https://huggingface.co/Rostlab/prot_bert
LCPBert	This paper	https://github.com/xinguoguo9211/LCPBert_Project

### Experimental model and study participant details

#### Ethics statement

Ethical approval for this study was granted by the Institutional Review Boards of the participating hospitals (approval numbers: SCCHEC-02-2021-037 and SIAT-IRB-240915-H0908) prior to study initiation. The study was conducted in accordance with the Declaration of Helsinki. Written informed consent was obtained from all participants before enrollment and sample collection. All experimental procedures and personnel training complied with the relevant guidelines and regulations of the participating institutions.

#### Study cohorts

A total of 6,705 samples were collected and categorized into nine cohorts: HD_0, which encompassed 2,199 peripheral blood samples from healthy donors and was designated for model construction; LC_0, consisting of 2,860 peripheral blood samples from early-stage lung cancer patients and also intended for model construction; HD_1, serving as the internal validation cohort with 500 peripheral blood samples from healthy donors (randomly selected from a pool of 2,699 peripheral blood samples from healthy donors, HD_0 + HD_1); BPN, comprising 255 peripheral blood samples from patients with benign pulmonary nodules and acting as part of internal validation cohort; LC_1, serving as the internal validation cohort with 500 peripheral blood samples from early-stage lung cancer patients (randomly selected from 3,360 peripheral blood samples from early-stage lung cancer patients, LC_0 + LC_1); HD_2, serving as the external validation cohort with 60 peripheral blood samples from healthy donors; LC_2B, representing 81 peripheral blood samples from patients with early-stage non-small cell lung cancer (NSCLC) and included in the external validation cohort; LC_2TIL, denoting 166 tumor-infiltrating lymphocyte (TIL) samples from patients with NSCLC and also part of the external validation cohort; LC_2T, including 46 tumor tissue samples from NSCLC patients and serving as a component of the external validation cohort. Additionally, the longitudinal cohort LC_SBRT comprised 38 peripheral blood samples from 19 NSCLC patients, collected at matched time points both pre- and 24 hours post-stereotactic body radiation therapy (SBRT). A summary of cohort characteristics is provided in Table 1, with further details in Table S6. Information includes sample ID, age, disease type, cell source, and data source, among other variables. Sex had no significant influence on the study results. Gender was not analyzed (a limitation due to lack of data collection).

### Method details

#### Study design

This study aimed to develop a diagnostic scoring system for lung cancer based on peripheral blood T-cell receptor (TCR) repertoire data. To achieve this, we developed the LCPBert framework, a trained protein language model leveraging the pre-trained ProtBERT model, to identify early-stage lung cancer-associated TCR CDR3 (complementary determining region 3) within the repertoire. The Lung Cancer Risk Index (LCRI), derived from the LCPBert framework, was then used to assess the lung cancer status of individual patients.

#### TCR β-chain sequencing

All lung cancer and control samples were processed using identical extraction and sequencing protocols and were randomly pooled during library preparation and sequencing. TCR β-chain sequencing was performed using DNA extracted from tumor and peripheral blood mononuclear cell samples following a multiplex PCR approach.^21^

Each sample library was sequenced on Illumina Novaseq platform, generating an average 2Gb raw data per sample. Then Raw FASTQ data was trimmed and processed using FASTP (v0.19.7)^38^ to remove adapters and low-quality reads. TCR repertoires were profiled from merged sequences using MiXCR (v3.0.4)^39^ by aligning against human TCRβ gene segments in IMGT database (https://www.imgt.org), and assembled TCR clonotype data were exported. VDJtools (v1.2.135)^40^ was used to convert the TCR clonotype files to a VDJtools-compatible format. For quality control, TCR data were excluded from further analysis if they failed to meet the following criteria: (1) Raw data yield ≥ 2GB; (2) > 85% of reads contained V/J primers; (3) > 70% of sequenced reads contained CDR3-aligned sequences.

#### TCR repertoire diversity analyses

The CDR3 amino acid (CDR3aa) sequences with stop codons or frameshifts were defined to be nonproductive (nonfunctional) amino acid sequences. For each sample, after excluding nonproductive TCR sequences, 100,000 random TCR gene sequences were selected for subsequent analyses. We assessed TCR repertoire diversity using three established metrics: D50, Shannon index, and Simpson index. Higher values for these indices correspond to greater diversity. D50 was defined as the minimal proportion of unique TCR CDR3 sequences required to account for 50% of the cumulative TCR sequence abundance in a sample. Additionally, high-expansion clones (HECs) were defined as those with a frequency exceeding 0.5% of the total TCR repertoire. The Shannon diversity index and Simpson diversity index were calculated using the following formulas respectively:

(Equation 1) $$\text{Shannon}\;\text{index}=-\sum_{i=1}^{n}fi\times lnfi$$

(Equation 2) $$\text{Simpson}\;\text{index}=1-\sum_{i=1}^{n}{fi}^{ˆ2}$$

(Where *fi* is the frequency of clone *i* and n is the total number of clones).

#### Lung cancer risk index (LCRI)

The Lung Cancer Risk Index (LCRI) was defined as the sum of the expansion frequencies of all TCRs identified as cancer TCRs within the individual. LCRI value ranged from 0 to 1 to evaluate individual lung cancer risk, with higher values indicating a greater risk. The formula of the LCRI was as follows.

(Equation 3) $$\text{Lung}\;\text{Cancer}\;\text{risk}\;\text{index}=\sum_{i=1}^{n}fi$$

(Where *fi* represents the clonal frequency of a specific TCR clone. A “lung cancer-associated TCR” is defined as a clone whose model-predicted probability of association exceeds the optimal, CDR3 length-specific discrimination threshold.).

#### LCPBert framework workflow

##### Data processing

Non-lung cancer CDR3 were derived from 2,199 healthy donors through: (1) selection of TCR CDR3 sequences with frequency >0.03% (CDR3 length 11-20 aa); (2) consolidation with duplicate removal; (3) CD-HIT^41^ clustering (RRID:SCR_007105; parameters: -c 0.4, -n 2, -T 4, -M 1500); (4) retention of all unique sequences from clusters (≥15% representation). This yielded 300,140 non-lung cancer unique TCR CDR3 sequences.

Lung cancer-associated CDR3 were obtained from 2,860 patients via: (1) selection at >0.04% frequency (CDR3=11-20 aa); (2) exclusion of sequences presented in healthy donors; (3) CD-HIT clustering with identical parameters; (4) extraction of all unique sequences from dominant clusters (≥15% frequency). This generated 261,579 lung cancer-associated unique TCR CDR3 sequences.

#### LCPBert model construction

LCPBert model comprises three modules. In the input phase, CDR3 sequences spanning 11 to 20 amino acid residues were partitioned by length. For each length group (L=11 to 20), amino acids were tokenized into unique integer indices. These indices were transformed into fixed-dimensional embedding vectors through an embedding layer, serving as input to length-specific ProtBERT models.

In the fine-tuning phase of LCPBert, we leveraged ProtBERT,^42^ a deep bidirectional transformer specifically engineered for protein sequence representation. Diverging from standard BERT architectures, ProtBERT scales to 30 layers to capture complex long-range dependencies inherent in amino acid chains. The model was self-supervised pre-trained on two complementary databases: UniRef100,^43^ providing evolutionarily refined reference sequences, and BFD,^44^ an integrated resource combining UniProt^45^ canonical sequences with metagenomic translations, collectively spanning over 200 million protein sequences. To adapt ProtBERT for lung cancer-associated T cell receptor (TCR) recognition, we performed task-specific fine-tuning. The training dataset comprised TCRβ sequences labeled as lung cancer-associated (1) or non-lung cancer (0). This binary classification framework enabled the model to learn discriminative features directly from primary TCR sequences, leveraging ProtBERT’s capacity to decode contextual patterns and functional motifs through residue-level embeddings.

The fine-tuning parameters were dynamically adjusted according to the CDR3 length to optimize performance. Specifically, the learning rate (LR) was set as follows: 3e-5 for CDR3 lengths of 11 and 12; 5e-6 for lengths 13, 14, 16, and 17; 3e-7 for length 15; 1e-5 for lengths 18 and 19; and 1e-6 for length 20. A linear learning rate scheduler was employed with a warmup ratio of 0.1. The per-device batch sizes for both training and evaluation were set to 32, and the model was trained for 5 epochs. Additional settings encompassed a weight decay of 0.01, a random seed of 42, and gradient accumulation steps of 1. The best model was selected based on accuracy metric, with the highest value indicating superior performance.

In the output phase, the output layer consists of two neurons, corresponding to the class labels of lung cancer-associated TCRs and non-lung cancer TCRs. A Softmax activation function is applied at the output layer to convert the raw outputs (logits) into probability values, indicating the likelihood of an input belonging to each class. In the task of predicting lung cancer-associated TCR CDR3, determining the optimal classification threshold is critical. We employed Receiver Operating Characteristic (ROC) curve analysis to identify this threshold. This approach was chosen because the dataset contained a greater number of TCRs derived from non-lung cancer sources compared with those from lung cancer, a potential source of bias that could affect prediction accuracy if not addressed.

### Quantification and statistical analysis

LCPBert was developed using Python 3, with model construction leveraging the GPU-enabled PyTorch framework (version 1.12.1). The multivariate regression model and the likelihood ratio test were analyzed using Python. The remaining statistical analyses and data visualization were performed using R, the statistical programming language (version 4.2.3). Mann-Whitney U test was used to compare differences between two groups, ∗, *p* < 0.05, ∗∗, *p* < 0.01, ∗∗∗, *p* < 0.001. A p-value < 0.05 was considered statistically significant. Prediction model performance was evaluated by using the area under the curve (AUC) based on the ROC curves.

## Supplemental information

Document S1. Figures S1 and S2 and Tables S1 and S3–S5

Table S2. 195 distinct VJ pairs between the HD and the LC

Table S6. Summary statistics for the study cohorts
