# Supplementary material for: LCPBert: ProtBERT-based early-stage lung cancer prediction from T cell receptor beta sequences
Source: iScience. 2026 Apr 1;29(5):115581. doi: 10.1016/j.isci.2026.115581 (PMC13098609; doi:10.1016/j.isci.2026.115581)
Supplement: Document S1. Figures S1 and S2 and Tables S1 and S3–S5 [file mmc1.pdf]

**iScience, Volume *29***

## Supplemental information

# LCPBert: ProtBERT-based early-stage lung cancer prediction from T cell receptor beta sequences

**Xin Yang, Yuwei Zhou, Zixuan Zhang, Huaichao Luo, and Jian Huang**

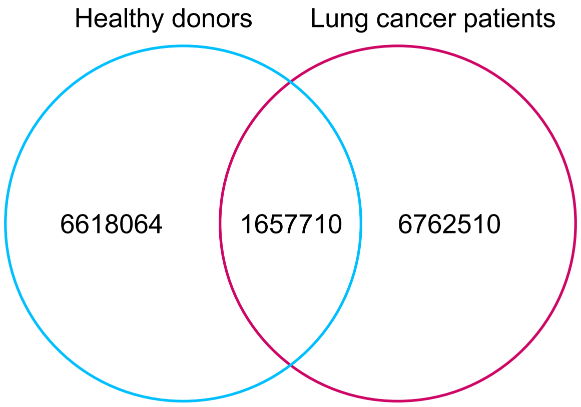


**Figure S1**. Venn diagram illustrating overlap of TCR clones between non-lung cancer and lung cancer-associated TCRs

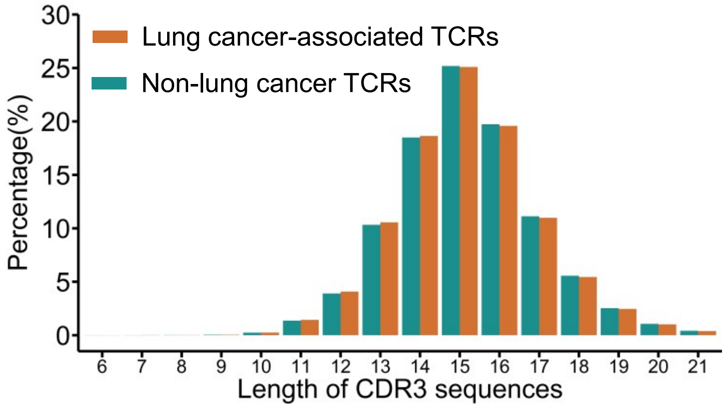


**Figure S2**. Length distribution chart of lung cancer-associated sequences and non-lung cancer sequences

**Table S1.** 14 distinct Vβ genes and 1distinct jβ gene between the HD and the LC

| Vβ/Jβ | LC_mean/HD_mean | LC_mean-HD_mean | p-value | p.signif |
|---|---|---|---|---|
| **TRBV3-1** | 1.431935 | 0.009579446 | 6.00E-253 | *** |
| **TRBV4-1** | 1.348376 | 0.007113303 | 8.38E-157 | *** |
| **TRBV4-2** | 1.36169 | 0.00358772 | 3.90E-129 | *** |
| **TRBV4-3** | 1.621249 | 0.004609472 | 0.0000028 | *** |
| **TRBV6-2** | 0.727268 | -0.011413923 | 8.04E-211 | *** |
| **TRBV6-3** | 0.384238 | -0.000112231 | 1.94E-154 | *** |
| **TRBV7-4** | 0.256576 | -0.001145539 | 0 | *** |
| **TRBV7-7** | 0.766718 | -0.000709725 | 1.17E-158 | *** |
| **TRBV10-3** | 0.748835 | -0.005405741 | 0 | *** |
| **TRBV18** | 1.491936 | 0.00756468 | 0 | *** |
| **TRBV24-1** | 0.745106 | -0.004372062 | 2.86E-243 | *** |
| **TRBV27** | 2.565203 | 0.011767668 | 0 | *** |
| **TRBV28** | 0.590003 | -0.040854608 | 0 | *** |
| **TRBV30** | 1.415172 | 0.006841344 | 1.89E-188 | *** |
| **TRBJ1-5** | 0.758385 | -0.00925349 | 6.80E-238 | *** |

**Table S3.** Distribution of sequence counts for Non-lung cancer and Lung cancer-associated CDR3 in Training, Validation, and Test sets

| **Length （aa）** | | **11** | **12** | **13** | **14** | **15** | **16** | **17** | **18** | **19** | **20** |
|---|---|---|---|---|---|---|---|---|---|---|---|
| **Non-lung cancer CDR3** | **Train** | 982 | 6919 | 17641 | 43988 | 76490 | 48617 | 23888 | 10989 | 4747 | 1777 |
| | **Valid** | 245 | 1692 | 4302 | 11270 | 19078 | 12106 | 6060 | 2743 | 1147 | 459 |
| | **Test** | 500 | 500 | 500 | 500 | 500 | 500 | 500 | 500 | 500 | 500 |
| **Lung cancer-associated CDR3** | **Train** | 577 | 2499 | 9221 | 29579 | 58316 | 53031 | 29927 | 13724 | 6057 | 2406 |
| | **Valid** | 154 | 637 | 2272 | 7301 | 14674 | 13279 | 7315 | 3414 | 1548 | 648 |
| | **Test** | 500 | 500 | 500 | 500 | 500 | 500 | 500 | 500 | 500 | 500 |

**Table S4. The LCRI of 19 patients before and after SBRT**

| File name | LCRI | Patient ID | Condition | Distant metastasis (DM) status |
|---|---|---|---|---|
| **SRR16122668** | 0.333803 | P01 | PreSBRT | Non-DM |
| **SRR16122670** | 0.229762 | P01 | PostSBRT | Non-DM |
| **SRR16122679** | 0.282313 | P02 | PreSBRT | DM |
| **SRR16122680** | 0.305389 | P02 | PostSBRT | DM |
| **SRR16122671** | 0.151244 | P03 | PreSBRT | Non-DM |
| **SRR16122672** | 0.230115 | P03 | PostSBRT | Non-DM |
| **SRR16122700** | 0.443821 | P04 | PreSBRT | Non-DM |
| **SRR16122701** | 0.304838 | P04 | PostSBRT | Non-DM |
| **SRR16122687** | 0.08063 | P05 | PreSBRT | Non-DM |
| **SRR16122688** | 0.276893 | P05 | PostSBRT | Non-DM |
| **SRR16122689** | 0.233791 | P06 | PreSBRT | DM |
| **SRR16122690** | 0.402862 | P06 | PostSBRT | DM |
| **SRR16122683** | 0.080632 | P07 | PreSBRT | Non-DM |
| **SRR16122684** | 0.074166 | P07 | PostSBRT | Non-DM |
| **SRR16122698** | 0.203684 | P08 | PreSBRT | Non-DM |
| **SRR16122699** | 0.158925 | P08 | PostSBRT | Non-DM |
| **SRR16122685** | 0.22393 | P09 | PreSBRT | DM |
| **SRR16122686** | 0.386277 | P09 | PostSBRT | DM |
| **SRR16122667** | 0.086081 | P10 | PreSBRT | Non-DM |
| **SRR16122682** | 0.158113 | P10 | PostSBRT | Non-DM |
| **SRR16122681** | 0.2611 | P11 | PreSBRT | DM |
| **SRR16122693** | 0.285991 | P11 | PostSBRT | DM |
| **SRR16122702** | 0.268967 | P12 | PreSBRT | Non-DM |
| **SRR16122669** | 0.271485 | P12 | PostSBRT | Non-DM |
| **SRR16122691** | 0.29534 | P13 | PreSBRT | Non-DM |
| **SRR16122692** | 0.25491 | P13 | PostSBRT | Non-DM |
| **SRR16122675** | 0.268114 | P14 | PreSBRT | Non-DM |
| **SRR16122676** | 0.303798 | P14 | PostSBRT | Non-DM |
| **SRR16122665** | 0.112602 | P15 | PreSBRT | Non-DM |
| **SRR16122666** | 0.155315 | P15 | PostSBRT | Non-DM |
| **SRR16122696** | 0.172843 | P16 | PreSBRT | Non-DM |
| **SRR16122697** | 0.175704 | P16 | PostSBRT | Non-DM |
| **SRR16122694** | 0.227379 | P17 | PreSBRT | Non-DM |
| **SRR16122695** | 0.263172 | P17 | PostSBRT | Non-DM |
| **SRR16122677** | 0.110781 | P18 | PreSBRT | Non-DM |
| **SRR16122678** | 0.115837 | P18 | PostSBRT | Non-DM |
| **SRR16122673** | 0.329786 | P19 | PreSBRT | Non-DM |
| **SRR16122674** | 0.325683 | P19 | PostSBRT | Non-DM |

**Table S5. Multiple linear regression analysis of factors associated with TCR diversity (D50) in the internal validation cohort.**

| File name | Estimate | Std. Error | t value | Pr(>\|t\|) |
|---|---|---|---|---|
| **(Intercept)** | 0.2197867 | 0.0058294 | 37.703 | < 2e-16 *** |
| **Age** | -0.0010578 | 0.0001204 | -8.786 | < 2e-16 *** |
| **Lung cancer status** | -0.0172365 | 0.0035070 | -4.915 | 1.01e-06 *** |
